# Scheduling Framework for Accelerating Multiple Detection-Free Object Trackers

**DOI:** 10.3390/s23073432

**Published:** 2023-03-24

**Authors:** Myungsun Kim, Inmo Kim, Jihyeon Yong, Hyuksoo Kim

**Affiliations:** 1 Department of Applied Artificial Intelligence, Hansung University, Seoul 02876, Republic of Korea; 2 Department of IT Convergence Engineering, Hansung University, Seoul 02876, Republic of Korea

**Keywords:** GPU scheduling, object tracking, multi-DNN, multi-threading, detection-free tracker

## Abstract

In detection-free tracking, after users freely designate the location of the object to be tracked in the first frame of the video sequence, the location of the object is continuously found in the following video frame sequence. Recently, technologies using a Siamese network and transformer based on DNN modules have been evaluated as very excellent in terms of tracking accuracy. The high computational complexity due to the usage of the DNN module is not a preferred feature in terms of execution speed, and when tracking two or more objects, a bottleneck effect occurs in the DNN accelerator such as the GPU, which inevitably results in a larger delay. To address this problem, we propose a tracker scheduling framework. First, the computation structures of representative trackers are analyzed, and the scheduling unit suitable for the execution characteristics of each tracker is derived. Based on this analysis, the decomposed workloads of trackers are multi-threaded under the control of the scheduling framework. CPU-side multi-threading leads the GPU to a work-conserving state while enabling parallel processing as much as possible even within a single GPU depending on the resource availability of the internal hardware. The proposed framework is a general-purpose system-level software solution that can be applied not only to GPUs but also to other hardware accelerators. As a result of confirmation through various experiments, when tracking two objects, the execution speed was improved by up to 55% while maintaining almost the same accuracy as the existing method.

## 1. Introduction

In a wide range of AI (artificial intelligence)-enabled service fields such as human–computer interaction [1], traffic control [2], video surveillance [3], and augmented reality [4], object-tracking technology has drawn constant attention. Object tracking is largely divided into detection-free tracking and tracking-by-detection. Recent studies have used tracking-by-detection methodologies to realize MOT (multi-object tracking). However, this is a method of tracking classified objects in advance and the process of revealing the association between the detection results. Detection-free tracking, which allows users to track any object from the user point of view, can be a more useful technology for security and safety-related applications such as crime prevention and facility safety. VOT (visual object tracking) is a kind of detection-free tracking, which estimates the position of the user-defined target object in a series of video frames. In doing so, the estimated position in each frame is usually defined by the bounding box including the target object to be tracked.

Without loss of generality, in order to secure the improved inference accuracy, DNN (deep neural network) models are getting bigger and more complicated [5]. Trackers with the latest technologies are also equipped with DNN models, and the computational complexity is also very high [6,7]. Therefore, tracking two or more user-specified objects in a video frame is even more computationally complex and makes the system very slow.

The recent trend of VOT technology can be divided into Siamese-network-based and transformer-based studies. Siamese network structure tracks target object by computing the similarity between the target patch designated by the user and the search region of the video frames [6]. Transformer-based trackers conduct tracking by fusing the features of the target patch and search region of the frames using attention mechanism [7]. These two kinds of trackers have very different structures. Therefore, using the optimization technique of the same method is not meaningful to make up for the speed-performance deterioration incurred when tracking two or more objects.

In order to maximize the execution speed of DNN, hardware accelerators specialized in specific DNN modules have been released [5]. However, these specialized accelerators do not guarantee their performance even in the new DNN structure. Therefore, edge devices and servers usually use GPUs to accelerate DNN modules. The DNN modules used in object trackers are also dependent on the GPU-specific libraries used by deep learning frameworks such as TensorFlow [8] and PyTorch [9], and the libraries are not optimized for various kinds of GPU hardware structures. Furthermore, these deep learning frameworks do not provide optimization techniques for two or more DNN modules to run parallel in the GPU even when the GPU is experiencing under utilization, which may lead the tracker performance to be suboptimal.

To tackle the above-mentioned issues, we propose a software-based solution approach, which provides an efficient scheduling framework for the two well-performing object trackers running on edge devices and GPU-server computing systems. We first lay the groundwork for the proposed scheduling framework to optimally map workloads included in the tracker to computing units. To this end, an in-depth computational structure analysis is conducted on SiamRPN++, which epitomizes Siamese-network-based trackers, and CSWinTT, which best exemplifies transformer-based trackers. Particularly, we give most of our attention to the large-scale computational structure of MHA (multi-head attention), which transformer-based trackers have in common, from the DNN module perspective.

Second, the proposed scheduling framework improves the tracking speed of two or more trackers when they are running together. This means that the tracking performance is improved when two or more objects are simultaneously tracked in a detection-free manner. The proposed scheduling framework is a system-level acceleration technology designed to be independent of the different structures of GPUs. Additionally, the approach proposed in this study can be applied to hardware accelerators other than GPUs. This is possible with only a library provided by the accelerator manufacturer.

## 2. Background and Related Work

To provide an aid in understanding the remaining part of this paper, this section gives background knowledge and related object tracking studies that have been previously conducted. In addition, the two types of trackers targeted in this paper are technically described.

### 2.1. Object Tracking

Object tracking refers to the process of estimating the position of an object or several objects that move over time in video frames, and generally, object tracking is divided into two categories depending on employed tracking algorithms: VOT (visual object tracking) and MOT (multiple object tracking). Object tracking typically outputs a bounding box, which has the location information of the object in each video frame [6,10,11].

VOT tracks a single object and class-agnostic. In VOT, only the position (i.e., bounding box) of the object in the first frame is given without any other information. There is no detailed information about the object, but as long as the location information of the object in the first frame is provided, the object can be tracked continuously in consequent video frames. VOT falls under the category of detection-free tracking, which means that a manually initialized bounding box is required for the tracking target rather than the detection of the predefined target object.

Unlike VOT, MOT tracks objects in predetermined classes. MOT automatically identifies multiple objects in a video and shows them as a series of trajectories. MOT tracks multiple objects and is commonly known as detection-based tracking, thus performing object detection every frame and associating the results with tracking. In other words, connecting the detected location information of the current frame with the one of the previous frame. For example, if there is a video of several cars driving on the road, MOT tracks each car separately.

### 2.2. Detection-Free Tracking

Our target tracking systems are detection-free trackers, and thus the trackers in this paper aim to keep track of multiple objects designated by the user in the first frame with VOT as the default mechanism, not MOT. Representative trends of trackers using VOT technology are based on either the Siamese network or the transformer architecture.

#### 2.2.1. Siamese-Network-Based Trackers

SiamFC [10] is a seminal study using a Siamese network for object tracking. A user creates an exemplar image *z* including a tracking target, and search image frames *x* means video frames that need to be inferred. *x* and *z* pass through the same CNN and their output tensors become the input of the cross-correlation operation. Thereafter, each component of the calculated similarity map corresponds to the similarity with *z* with respect to the inside *x*. The SiamRPN [11] adopts an RPN (region proposal network), which was used as a standard in image detection problems, in SiamFC, and performs bounding box regression to determine the location of tracked target. As a result, the size of the tracked object can be estimated more accurately than before, and at the same time, the iterative calculation due to the adoption of the image pyramid can be avoided. As a way to solve the problem of decreasing accuracy by padding inside CNN, SiamRPN++ [6] proposes a spatial-aware-sampling strategy to make the locations of tracked objects in the search image frame have a uniform distribution. In fact, by applying learning data collected by the strategy, a SiamRPN tracker that adopted ResNet-50 [12] as a backbone obtains a higher accuracy than a SiamRPN with AlexNet [13].

#### 2.2.2. Transformer-Based Trackers

Transformer-based approaches have drawn great performance in various AI applications such as object detection, semantic segmentation, and image recognition. The success factors in such fields are from the fact that a cross-attention mechanism enables relevant reasoning between image patches [14]. Even in object tracking research works, transformer-based trackers have presented their excellent achievements by incorporating the pixel-level attention to mingle the features of the target object and tracked object in search image frames. TransT [15] introduces an attention mechanism to perform feature fusion of target object and search image frames. The designed feature fusion network is structured with two modules: ECA (ego-context augment) module and CFA (cross-feature augment) module. The two modules expedite bounding box regression and object localization. STARK [16] suggests an encoder–decoder structured transformer which takes both spatial and temporal information into account. The encoder with self-attention modules learns the relationship between the target object and the incoming video frames by analyzing feature dependencies. To achieve target position estimation, the decoder learns a query embedding. Swin Transformer [17] takes up a hierarchical structure and consists of transformers. To obtain an expanded receptive region, it gradually increases the size of image patches. CSWinTT [7] develops pixel-level attention into window-level attention while inheriting the structural advantage of Swin Transformer. Cyclic shifting has the effect of expanding the window area, which greatly improves the accuracy.

### 2.3. Structural Analysis of Detection-Free Object Trackers

In this subsection, we provide a concise description of the architecture of two representative detection-free object trackers.

#### 2.3.1. SiamRPN++: Siamese-Network-Based

**Figure 1:**
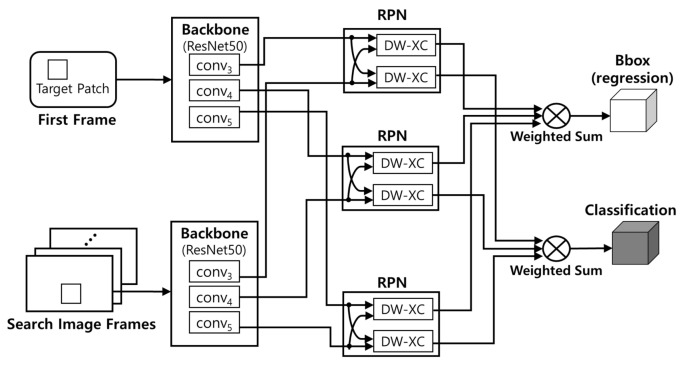
Workflow of SiamRPN++.

Figure 1 shows the overall workflow of SiamRPN++. For input data, the target patch with the object to be tracked and the video frames are used, and the video frames are generally called as search region or search image frames. In the first frame, the user sets the location information of the target patch containing the object to be tracked. The location information of the target patch consists of a total of four values, given the *x* and *y* coordinate values at the upper left, and the values of width and height based on them.

The target patch and search image frames are transmitted to ResNet-50-based backbones. Each backbone outputs three different feature maps; then, they are inputted to three RPN (region proposal network) blocks to perform a similarity check. To do so, two identical DW-XC (depth-wise cross-correlation) modules are applied [6]. Through the weighted sum operation, the three bounding box values and classification results from the three RPN modules derive one bounding box regression and a classification result, which are the final results of inference. The closer the value of classification result is to one, the higher the probability that the object is in the bounding box.

Equation (1) details the two weighted sum procedures shown in Figure 1. $S_{all}$ and $B_{all}$ represent the final classification and regression results, respectively [6]. $S_{l}$ and $B_{l}$ represent the classification and regression of each RPN, and *l* is one of 3, 4, and 5, indicating that it is the result from conv3, conv4, and conv5 in backbones. $\alpha_{i}$ and $\beta_{i}$ are combination weights, and they are obtained after offline end-to-end optimization [6].

(1) $$S_{all}=\sum_{l=3}^{5}\alpha_{i}*S_{l},\;B_{all}=\sum_{l=3}^{5}\beta_{i}*B_{l}.$$

#### 2.3.2. CSWinTT: Transformer-Based

**Figure 2:**
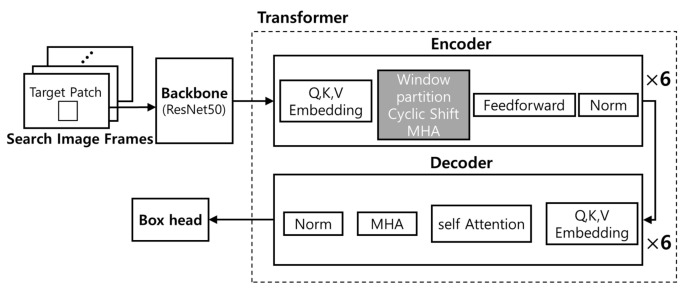
Workflow of CSWinTT.

Basically, just as SiamRPN++ explained above, CSWinTT also receives the target patch and search image frames while using just one ResNet-50 backbone. Overall, it is constructed very similar to general transformer-based object trackers [14,15,16,17] and has a large computational complexity. CSWinTT has six-layer encoder and decoder blocks, and each encoder and decoder has MHA (multi-head attention). The core technology of CSWinTT centers around MHA incorporating window-partitioning and cyclic shifting, and the gray box in Figure 2 denotes it.

CSWinTT also sets the bounding box position as the final result and outputs confidence score every frame along with the bounding box. Confidence score is the probability of the presence or absence of the target object to be found in the bounding box, which is the result of inference about every single frame. The closer it is to the value of one, the higher the probability that the object is in that bounding box.

## 3. Problem Settings

In this paper, we aim to show a mechanism that maximizes the execution speed while maintaining inference accuracy when multiple detection-free-based trackers are running on edge device or GPU-server systems to track multiple objects. There are some difficulties that must be noted in such an execution environment.

- Systems that run DNN models, such as embedded edge devices and server systems, are typically composed of many CPUs and a much smaller number of DNN accelerators. Thus, multiple tracker tasks hosted by the CPUs can throw DNN workloads required for object tracking into the accelerator independently of each other. In this situation, the performance of trackers may differ greatly depending on the scheduling policy imposed on DNN workloads delivered to the accelerator.
- For instance, a GPU is a representative DNN accelerator. As shown in Figure 1 and Figure 2, backbone, RPN, and MHA blocks have different computational complexity. Here, the GPU is not always 100% used depending on which block is computed. For example, when there are two DNN workloads that occupy 30% of GPU utilization, they maintain 30% utilization if they are performed in order in a row. However, if the two workloads are on the GPU at the same time, they can have twice the execution speed with 60% utilization. However, it is not easy to double GPU utilization under real-world applications. Libraries such as cuDNN [18] and CUDA runtime [19] do not adaptively allocate DNN workloads to all different GPU hardware architectures.
- Since multiple CPUs are supported, multiple trackers may allocate DNN workloads to the accelerator such as the GPU. Moreover, with the help of commercial DNN frameworks such as TensorFlow [8] and PyTorch [9], we can easily design trackers using Python. However, if hardware blocks such as MPS (multi-process service) [20] are not supported in an embedded environment, occurred overhead is unavoidable due to the context-switching, where context means the virtual address boundary of processes. In addition, even if multi-threading is available within the same context (process), it is difficult to avoid the serialization problem caused by the GIL (global interpreter lock) policy of Python.
- SiamRPN++ and CSWinTT have very heterogeneous structures, as shown earlier in Figure 1 and Figure 2. Thus, a uniform DNN workload scheduling scheme can lead to poor performance for some trackers in a way that can benefit some trackers.

The problem we want to solve is providing an effective software-based means to get out of the difficulties listed above.

## 4. Solution

In this section, to solve the aforementioned problems, we explain our solution approach with sufficient technical details. First, the overall solution architecture is given, and then workload scheduling techniques and parallelization methods preferable to the execution characteristics of each tracker are described in detail.

### 4.1. Overall Solution Approach

**Figure 3:**
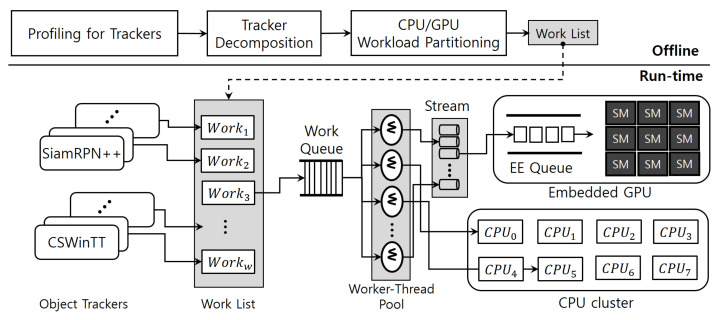
Overview of the scheduling framework for accelerating multiple detection-free object trackers.

Figure 3 details the operational workflow of the proposed solution approach in this paper. Roughly, the approach consists of offline and run-time phases. Offline, first, the execution time of each function block constituting the target tracker is measured through profiling. Then, a basic scheduling unit is derived by comprehensively considering this result and the data dependency between each functional block. In Figure 3, each work in the work list becomes an instance scheduled on the CPU (i.e., scheduling unit), and the figure shows an example of eight CPUs and *w* works. When each scheduling instance is obtained, a computing unit suitable for each instance, either a CPU or a GPU, is defined. Finally, the work list to be executed at run-time phase is completed according to this offline procedure.

In the run-time phase, multiple works in the work list are executed over the solution architecture, which consists largely of an offline defined work list, a work queue, and a worker-thread pool. A work $Work_{i}$ defined in the work list can be edited on a variety of scales. Particularly, it is possible from a small layer of a DNN model to the entire tracker. For example, $Work_{0}$ is the tracker task itself, and $Work_{1}$ and $Work_{2}$ are DNN workloads allocated by the tracker $Work_{0}$ to the GPU. First, $Work_{0}$ is mapped to one of threads in the worker-thread pool, and then $Work_{0}$ assigns $Work_{1}$ and $Work_{2}$ to the threads in the pool.

Object trackers can request their works ($Work_{0},Work_{1},\cdots ,Work_{7}$) asynchronously to the work queue. One of threads in the worker-thread pool immediately extracts the work at the queue front of the work queue whenever it is in the idle state. Then the worker thread first determines whether the delivered work is GPU-side or CPU-side. If the work is a DNN workload it is mapped to one of the streams [5,21], and then enqueued to the EE (execution engine) queue of the GPU [5,21], if not, it is assigned to one of the CPUs directly.

### 4.2. Scheduling Works in the Work List

The detailed operation of each function block shown in Figure 3 is explained through Algorithm 1. In the algorithm, the function **worker_thread( )** is the pseudo code of each worker thread. Both **queue_pull( )** and **queue_push( )** are provisioned as tools for accessing the work queue, and **queue_pull( )** is only called by worker threads and **queue_push( )** is utilized by each work, i.e., work in the algorithm.

When the proposed scheduling framework starts, the all threads within the worker-thread pool wait indefinitely since there is no work in the work queue. Then, offline defined works in the work list arrive at the work queue through the function **queue_push( )**. At this point in time, sig is broadcasted to all the threads in the worker-thread pool. Then, one of threads in the pool receives sig and exits the blocked state, as shown in line 3. Immediately after this, the function **queue_pull( )** is used, and the work (work) at the front of the work queue is transferred as an argument to the function **execute( )**. Looking at lines 13 and 14, **queue_pull( )** also broadcasts sig. This allows worker threads to take the next work after the first work is exited when there are two or more works in the work queue.
**Algorithm 1** Scheduling framework for multiple object trackers.1:**function** worker_thread( )2:    A:3:    wait_signal(sig)4:    work← queue_pull( )5:    execute(work)6:    goto A:7:**end function** 8:**function** queue_push(work)9:    insert work to the work queue10:    broadcast(sig)11:**end function** 12:**function** queue_pull( )13:    **if** (number of works in the queue > 1) **then**14:        broadcast(sig)15:    **end if**16:    **return** work at the queue front17:**end function** 18:**function** execute(work)19:    **if** (work is not a pure CPU workload) **then**20:        i← index of work21:        set the stream index as *i*22:    **end if**23:    **if** (work is a pure DNN workload) **then**24:        executing work25:        synchronize host until the DNN workload in the $i^{th}$ stream has completed26:        **return**27:    **end if**28:    executing work29:**end function**

The properties of the works in the work list can be expressed in three ways: pure CPU workloads, pure DNN workloads, and CPU–GPU mixed workloads. The parameter work passed to the function **execute( )** is one of these three. First, if it is not a pure CPU workload, it means that the GPU is used, so set the stream index for parallel processing in the GPU (lines 19 to 22).

If work is a pure DNN workload, work is transferred to the GPU’s EE queue and executed (line 24). At this time, synchronization must be performed with the host CPU for the next operation. For instance, let us say we have $Work_{1},Work_{2}$, and $Work_{3}$ in the work list. $Work_{1}$ and $Work_{2}$ are DNN workloads that use GPU, and Work3 needs to concatenate the operation results of $Work_{1}$ and $Work_{2}$. In this case, Work3 using the CPU must wait for the synchronization event that the GPU has finished all its assigned work (line 25).

In the case of a mix of CPU workload and DNN workload, work is simply executed as in line 28. At this time, the internal operation of work uses the GPU sporadically, but there is no synchronization process like in line 25. The reason for this is as follows. First, work is a decomposed internal tracker function. Therefore, in one function flow, the next workload that uses the CPU cannot be called until the GPU finishes its operation.

### 4.3. Execution Time Analysis for Decomposing Object Trackers

Table 1 and Table 2 represent execution time profiles for each functional blocks of SiamRPN++ and CSWinTT trackers, respectively. For measurement, we used one NVIDIA RTX A6000 GPU [22] and randomly extracted 3350 images from TrackingNet [23] as a dataset. As we can see from the two tables, CSwinTT is a very large-sized tracker that takes more than twice the time when dealing with 3350 images compared to SiamRPN++.

If we look at closely the time required for each functional block, the execution time of using DNN workloads (backbone and RPN) is shorter than that of others, where others in the tables include image loading and pre-processing. In other words, it has a short use of GPU. Therefore, it is not suitable to apply parallelism on DNN workloads, and because the execution time is short as a whole, small-sized subdivided works are not efficient. Too small-sized works can only cause scheduling overhead.

On the contrary, CSWinTT has a long overall processing time and has a long time to use DNN workloads (backbone, encoder, and decoder). In particular, the encoder block using DNN workloads accounts for almost 60% of the total execution time, so if we apply parallelism to this part, considerable performance gain can be expected.

### 4.4. Placement of Works Constituting SiamRPN++

**Figure 4:**
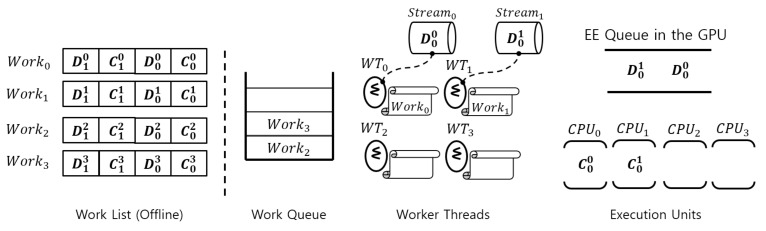
Snapshot of functional blocks in the proposed solution architecture through a walk-through example of SiamRPN++.

In Figure 4, we illustrate the operational flow of the proposed solution architecture with a walk-through example in a sequence of four works. As mentioned above, based on the computation analysis of SiamRPN++, each work is the entire SiamRPN++ tracker itself. $D_{j}^{i}$ denotes a DNN workload of $Work_{i}$ where *j* does the operational sequence index, and $C_{j}^{i}$ means for a CPU-side workload. In this example, as explained earlier, $D_{j}^{i}$ and $C_{j}^{i}$ are defined offline.

In the figure, the execution order of the DNN and CPU workloads inside the work list starts from the right to the left. $Work_{0}$ and $Work_{1}$ are already assigned to the worker-threads $WT_{0}$ and $WT_{1}$, respectively. Thus, $C_{0}^{0}$ and $C_{0}^{1}$, which are the first CPU-side workloads, are running on the two CPUs, and the following DNN workloads $D_{0}^{0}$ and $D_{1}^{0}$ are assigned to their streams. Note that in our design, each worker thread has its own dedicated stream; $Work_{i}$ has $Stream_{i}$. Through the streams, $D_{0}^{0}$ and $D_{1}^{0}$ are enqueued to the EE queue in the GPU, and depending on the SM (streaming multiprocessor) availability inside the GPU, $D_{0}^{0}$ and $D_{1}^{0}$ can be executed simultaneously. Since $Work_{2}$ and $Work_{3}$ are still in the work queue, these two works are not assigned to any of the two remaining worker threads $WT_{2}$ and $WT_{3}$. Once $WT_{2}$ pulls the queue from $Work_{2}$, $C_{0}^{2}$ of $WT_{2}$ takes $CPU_{2}$ and then starts to assign $D_{0}^{2}$ to $Stream_{2}$. Even on embedded edge devices with only one GPU, this capability allows multiple trackers to perform their tracking tasks in parallel.

### 4.5. Placement of Works Constituting CSWinTT

**Figure 5:**
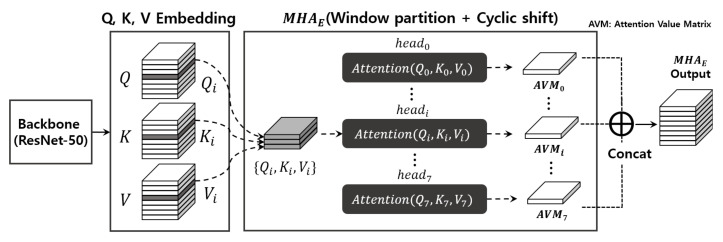
Workflow of multi-head attention in the encoder.

As shown in Table 2, the computational cost that the encoder block dominates in CSWinTT is substantial. On the basis of this profile data, before proceeding further, we closely analyze the encoder blocks with special focus on MHA blocks, which are commonly included in transformer-based DNN models. Figure 5 details multi-head attention $MHA_{E}$ performed inside the encoder block. The output of the backbone is converted into *Q* (queries), *K* (keys), and *V* (values) tensors through embedding. Each tensor consists of vectors as many as the number of heads and is represented as $Q=Concat\left(Q_{0},Q_{1},\cdots ,Q_{7}\right),K=Concat\left(K_{0},K_{1},\cdots ,K_{7}\right)$ and $V=Concat(V_{0},V_{1},\cdots ,V_{7})$, respectively. One of the heads $head_{i}$ takes $Q_{i},K_{i},V_{i}$ as input and outputs $i^{th}$ attention value matrix $AVM_{i}$ through $Attention(Q_{i},K_{i},V_{i})$ mechanism. Finally, the outputs of each transformer head ($AVM_{0},AVM_{1},\cdots ,AVM_{7}$) are concatenated together. To sum up, the final result of $MHA_{E}$ is obtained as below [7]:

(2) $$\begin{array}{c} MHA_{E}\left(Q,K,V\right)=Concat\left(AVM_{0},AVM_{1},\cdots ,AVM_{7}\right) \end{array}$$

(3) $$\begin{array}{c} where\;AVM_{i}=Attention\left(Q_{i},K_{i},V_{i}\right) \end{array}$$

(4) $$\begin{array}{c} =softmax\left(\frac{Q_{i}K_{i}^{T}}{\sqrt{d_{k}}}\right)V_{i} \end{array}$$

where $d_{k}$ means the dimension of key.

The point to note here is that each head has independent input, and thus all heads in $MHA_{E}$ can be executed in parallel and independently with each other by model parallelism [24]. Furthermore, as explained earlier, $MAH_{E}$ includes window partitioning and cyclic shifting, and has a high computational complexity that occupies about 58.7% of the total execution time. Accordingly, we can expect a significant reduction of CSWinTT execution time by processing all the heads in $MHA_{E}$ in parallel inside the GPU.

**Figure 6:**
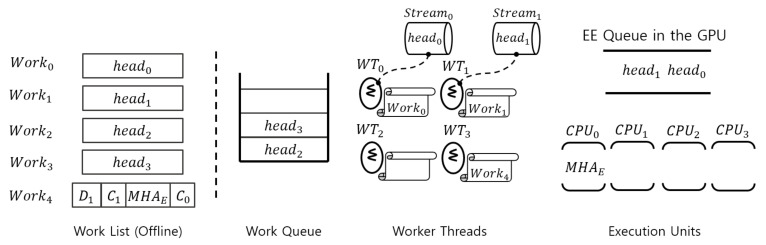
Snapshot of functional blocks in the proposed solution architecture through a walk-through example of CSWinTT.

For easy understanding, we explain how model parallelism applied to $MHA_{E}$ described above actually works through a figure. In Figure 6, we illustrate the operational flow of the proposed solution architecture with a walk-through example in a sequence of five works. Different from the case of SiamRPN++, based on the computation analysis of CSWinTT, a work can be the entire CSWinTT tracker itself or one of the transformer heads in MHA of the encoder. In the figure, $head_{i}$ means the $i^{th}$ transformer head and $MHA_{E}$ performs multi-head attention in the encoder.

To demonstrate the effect of model parallelism in the tracker, unlike the case of SiamRPN++, we just take an example of only one CSWinTT tracker. After the CPU-side workload $C_{0}$ finishes, $WT_{3}$ on $CPU_{0}$ performs $Work_{4}$. Next, $MHA_{E}$ brings both $Work_{0}$ and $Work_{1}$ one after the other from the work queue, assigning transformer heads $head_{0}$ and $head_{1}$ to their designated streams. Finally, both $head_{0}$ and $head_{1}$ are launched to the EE queue for parallel execution in side of the GPU.

An edge device such as NVIDIA AGX Xavier [25] has eight CPUs and one GPU, and CSWinTT uses eight transformer heads in $MHA_{E}$. Therefore, if we run one CSWinTT tracker on that device, Figure 6 is changed to have nine works and nine worker threads. In this case, all transformer heads ($head_{0}\;\;∼\;\;head_{7}$) are performed in parallel as much as possible, and then $C_{1}$ holds one CPU (possibly $CPU_{0}$) to process the obtained result parallelly executed by $head_{0}\;\;∼\;\;head_{7}$.

## 5. Experiments

In this section, we present the experimental verification we have conducted to validate the efficacy of the proposed solution approach. First, we explain implementation method and then elaborate on measurement results along with the relevant analysis.

### 5.1. Implementation Details

Basically, we take DNN modules built in both SiamRPN++ and CSWinTT trackers from the PyTorch framework [9]. All the threads in the worker-thread pool in Figure 3 are threads of the same process. This multi-threading scheme caters for several benefits in terms of scheduling management; all the functions assembled in all the worker threads are controlled under one single address space, providing the same synchronization primitives and truthful data sharing [5].

**Figure 7:**
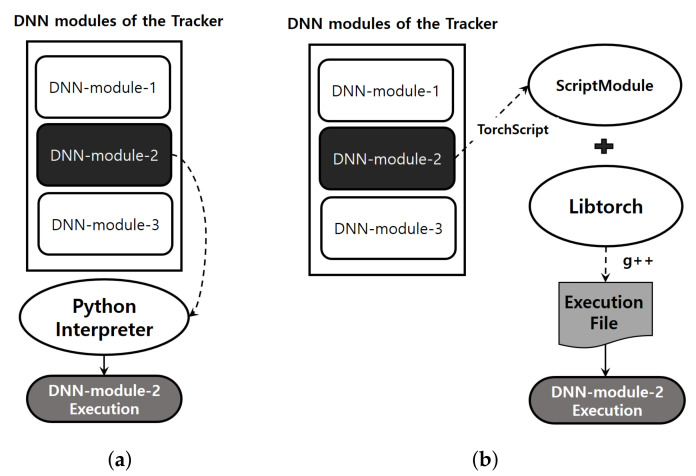
Comparing the execution environment: (**a**) existing mechanism for DNN module execution with Python interpreter and (**b**) proposed execution methodology.

DNN modules generated from PyTorch framework are made up of Python-based code, and each module is executed by the Python interpreter. In such an execution environment, GIL (global interpreter lock) enables only one thread to hold the access permission of the Python interpreter, preventing multiple DNN modules from running on several threads [26]. The higher the number of DNN workloads that launch the kernel to the GPU, the more work-conserving the GPU is, so CPU-side multi-threading is indispensable [21]. To clear up the innate constraints of GIL, we propose a new execution methodology and Figure 7 shows the before and after.

In our DNN execution method, to apply the C++-based execution environment, using TorchScript, DNN modules of the both trackers are changed into a ScriptModule [27]. After combining the libtorch library and ScriptModule, we compile it with a C++ compiler *g*++, and then obtain an executable file. Since using the compiled C++-programmed execution file is not controlled by GIL, multi-threaded programming is possible, and accordingly, multiple DNN workloads from DNN modules can be issued to the GPU at the same time.

### 5.2. Experimental Setup

For a more comprehensive verification, we adopt an edge device with limited computing resources and a server computing system, which is the opposite, as the target systems. We take the Jetson AGX Xavier platform [25] as the target edge device, and a GPU server equipped with 4 × NVIDIA RTX A6000 [22] for the target server computing system. The detailed both hardware and software specifications of the target systems are presented in Table 3 and Table 4.

As for workloads, TrackingNet [23] with 225,589 images is used. When the trackers are running on the edge device, only 3350 images, randomly selected, out of 225,589 total images of the TrackingNet dataset were used considering the storage space in the target edge device. Whereas for the GPU server, all images of TrackingNet were used.

To demonstrate the usefulness and practicality of the proposed solution approach under various conditions, all the experimental results are from the target edge device as well as from the target GPU server, and we diversified the validation methodologies suitable for each experimental stage. We basically evaluated the proposed solution approach against the original SiamRPN++ and CSWinTT trackers. In each graph, legend Org. denotes when the images from TrackingNet are processed by the original SiamRPN++ or CSWinTT trackers without any modifications, whereas Sol. is the case under our proposed solution approach.

We checked whether the approach we proposed is less accurate compared to the original trackers and observed how much it was contributing to the improvement of execution speed-up. As for the accuracy measurement, the evaluation metrics, area under the curve (AUC), precision (*P*), and normalized precision ($P_{normal}$) were adopted [7,23,28]. Given the ground truth bounding box ($BB^{gt}$) and the tracked one ($BB^{tr}$), the success score (i.e., overlap score) is defined as $S=\frac{|BB^{gt}\cap BB^{tr}|}{|BB^{gt}\cup BB^{tr}|}$, where ∪ and ∩ imply the union and the intersection of $BB^{gt}$ and $BB^{tr}$, respectively, and |·| means the number of pixels in that area [7,23,28]. The number of frames whose success score *S* is greater than the given threshold is measured. Using this number, the success plot is obtained to display the proportion of the success frames where the thresholds are ranged from 0 to 1. Ultimately, we can get AUC (area under the curve) from the success plot. The precision is defined as $P=\;∥C^{tr}-C^{gt}{∥}_{2}$, where $C^{tr}$ and $C^{gt}$ denote the centers of the tracker bounding box and the one of ground truth, respectively [7,23,28].

To confirm the speed improvement, in the case of edge devices, the time taken by trackers to process 3350 randomly selected images is compared through graphs, and in the case of GPU servers, the time to process all the images in TrackingNet is measured. In addition, the average frame per second (FPS) result is simultaneously presented on each experimental graph.

### 5.3. Experimental Results

#### 5.3.1. Inference Accuracy

As shown in Figure 7, DNN modules formed into the trackers are converted into ScriptModule. Then ScriptModule is compiled together with the libtorch library, creating an execution binary by the proposed execution environment. This may cause an inference accuracy gap compared to the existing interpreter-based method. Thus, to closely examine this, we measure the accuracy in terms of AUC,P, and $P_{normal}$.

Table 5 and Table 6 show the results from running SiamRPN++, and Table 7 and Table 8 are the cases for CSWinTT. Overall, comparing the accuracy of the tracker itself, it can be seen that the accuracy of CSwinTT is relatively excellent in both the previous execution environment and the proposed one.

When running SiamRPN++, we can see that the accuracy is slightly improved in three aspects in the proposed execution environment in both edge devices and GPU servers, but they are almost similar.

The case of CSWinTT showed the opposite result to the SiamRPN++ case. In the case of both edge device and GPU server, the proposed method showed a small accuracy drop. When running on the edge device, AUC decreased by 3.6, *P* by 6.3, and $P_{normal}$ by 2.576, indicating larger values than in the case of the GPU server.

#### 5.3.2. Inference Speed

Here, we report on the comparison result of the execution speed when two identical trackers are running simultaneously; multiple detection-free trackers are running together. The y-axis of all graphs means the time it takes for the tracker to track all the images used in the experiment. Therefore, the smaller the value, the higher the performance, and of course, the higher the FPS, the higher the execution speed.

**Figure 8:**
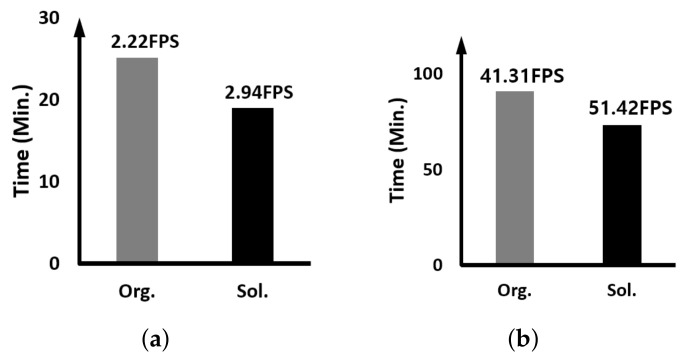
Comparing the execution time and speed when two identical SiamRPN++ trackers are running: (**a**) on the edge device and (**b**) on the GPU server.

Figure 8 displays the result when two identical SiamRPN++ trackers are running together. As can be seen in the figure, when our proposed approach is applied, the FPS increase rate is 32% and 24% on the edge device and GPU server, respectively. As can be seen in Table 1, the percentage of execution time occupied by the backbone and RPN, which are DNN workloads performed on the GPU, is smaller than that of time using the CPU, i.e., CPU dependence is relatively high. Compared with the GPU server, the difference in performance between the CPU and the GPU in the edge device is relatively smaller than that in the GPU server. Therefore, the execution speed improvement in the edge device is about 8% higher.

**Figure 9:**
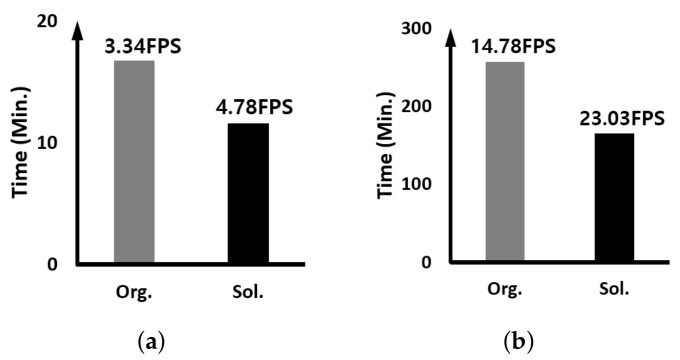
Comparing the execution time and speed when two identical CSWinTT trackers are running: (**a**) on the edge device and (**b**) on the GPU server.

Figure 9 shows the result when two identical CSWinTT trackers are running together. As we can see, when the proposed approach is applied, the FPS increase rate is 43% and 55% on the edge device and GPU server, respectively. This is the opposite result from the higher FPS increase rate on the edge device when we experimented with the SiamRPN++ tracker. As shown in Table 2, compared to SiamRPN++, CSWinTT has high computational dependence on the GPU, and MHA accounts for 58.7% of the total computation. Since the GPU server has a GPU with much better parallel processing capability than the embedded GPU of the edge device and the heads of $MHA_{E}$ use multi-threading and multi-stream, the FPS performance of the GPU server is remarkable.

**Figure 10:**
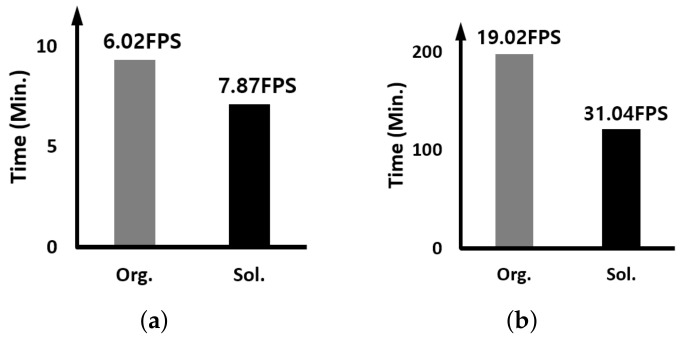
Comparing the execution time and speed when one CSWinTT tracker is running: (**a**) on the edge device and (**b**) on the GPU server.

Since $MHA_{E}$ of CSWinTT itself is parallelized, we verify that the proposed parallel scheduling technique works even when only one CSWinTT model is running. Figure 10 is the result of the experiment. As can be seen through the figure, the degree of FPS performance improvement is more noticeable when only one CSWinTT is running. This means that the case when the GPU embedded in the GPU server we target has 8 head operations of one CSWinTT is more effective compared to when 16 head operations of two CSWinTT trackers are mapped in parallel to SMs inside the GPU through the work queue.

**Figure 11:**
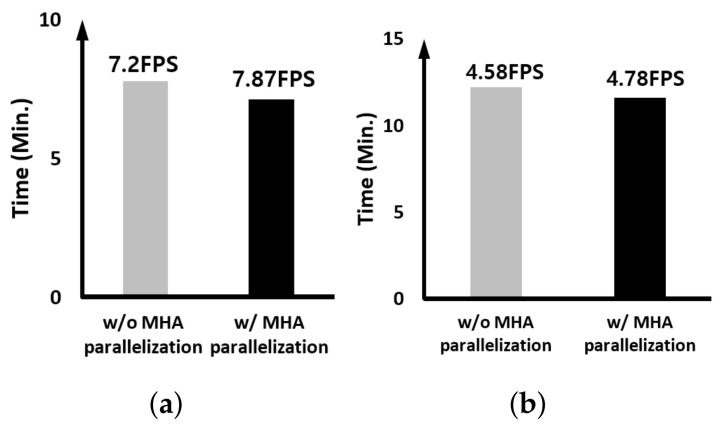
Comparing the MHA parallelization effect on the edge device: (**a**) 1× CSWinTT and (**b**) 2× CSWinTT.

**Figure 12:**
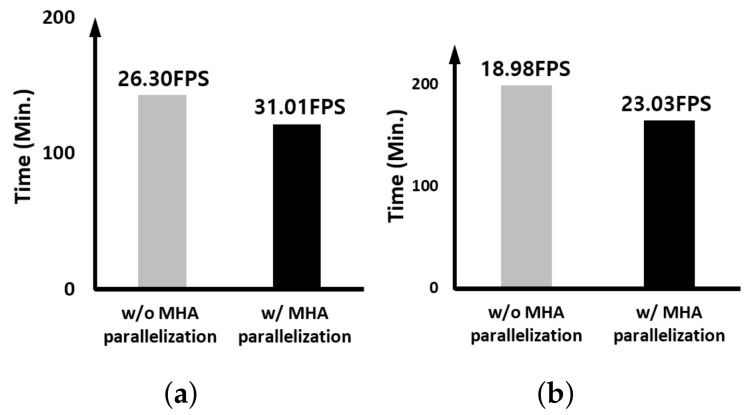
Comparing the MHA parallelization effect on the GPU server: (**a**) 1× CSWinTT and (**b**) 2× CSWinTT.

Next, only the effect when MHA is processed in parallel is verified, and Figure 11 and Figure 12 compare the results with and without the parallel execution of heads in MHA in the case of the edge device and the case for the GPU server, respectively. For the case of the edge device, the FPS increase rate is higher when only one CSWinTT tracker is executed compared to the case with two trackers. However, in case of GPU server, the result was the exact opposite. This result implies that if the internal hardware resource of the GPU is sufficient to perform multiple head operations in parallel, the effect of MHA parallel processing using CUDA stream can be maximized. However, on the edge device using the GPU with the limited hardware resource, the overall effect of multi-threading rather than MHA parallelization is more significant.

## 6. Conclusions

Object tracking technology is widely used in areas such as crime prevention, facility safety, traffic control, and information collection. Especially, detection-free object tracking technology that can track objects that are not of a predefined class has been highlighted as crucial in these applications. In this paper, we presented a framework that efficiently schedules the workloads inside detection-free trackers to work out the computing-related issues that occur when two or more detection-free-tracking tasks are running simultaneously. To achieve this, first, the computational structures of the Siamese-network-based tracker and the transformer-based tracker, which exhibit excellent tracking performance, are analyzed, and a scheduling unit suitable for each tracker is determined offline. At run-time, multi-threading allows trackers to use multiple CPUs concurrently, delivering multiple DNN workloads included in trackers to the GPU at the same time. By doing so, the GPU is kept work-conserving. As a result of experimental validation, when tracking two user-specified objects, the proposed scheduling framework led to a 55% performance improvement without reducing tracking accuracy. 

## Figures and Tables

**Table 1:** Execution time profile of SiamRPN++.

	Backbone	RPN	Others	Total
**Exe. Time (s)**	22.7	8.87	37.13	68.7
**Ratio**	33.0%	12.9%	54.1%	100%

**Table 2:** Execution time profile of CSWinTT.

	Backbone	Encoder	Decoder	Others	Total
**Exe. Time (s)**	22.6	100.69	11.41	36.83	171.53
**Ratio**	13.1%	58.7%	6.7%	21.5%	100%

**Table 3:** Specification of the target edge device.

	Classification	Description
HW	CPU	8-core ARM v8.2 Carmel 64-bit CPU, 8 MB L2, 4 MB L3 cache
	GPU	512-core Volta GPU with Tensor cores
	Memory	32 GB 256-Bit LPDDR4x, 137 GB/s
	Storage	32 GB eMMC 5.1
SW	Kernel Ver.	Linux 4.9.140
	SW Package	JetPack 4.2
	CUDA Ver.	CUDA v10.2

**Table 4:** Specification of the target GPU-server computing system.

	Classification	Description
HW	CPU	16-core, 64 MB L3 cache, 3.9 GHz
	GPU	NVIDIA RTX A6000, 336 Tensor Cores, 10,752 CUDA Cores,
		48 GB Memory, 309.7 TFLOPS
	Memory	4 × 64 GB DDR4 PC4
	Storage	1 × SSD 1.92 TBG 2.5” SATA
SW	Kernel Ver.	Linux 5.15.0
	SW Package	MPI Horovod, NVIDIA GPU Monitoring SW
	CUDA Ver.	CUDA v11.6

**Table 5:** SiamRPN++ running on the target edge device.

	AUC	*P*	$P_{\mathrm{normal}}$
**Org.**	77.19	78.1	88.97
**Sol.**	78.56	80.45	90.32

**Table 6:** SiamRPN++ running on the target GPU server.

	AUC	*P*	$P_{\mathrm{normal}}$
**Org.**	60.92	58.495	70.49
**Sol.**	63.65	61.9	73.57

**Table 7:** CSWinTT running on the target edge device.

	AUC	*P*	$P_{\mathrm{normal}}$
**Org.**	93.32	96.82	97.51
**Sol.**	93.72	90.52	94.93

**Table 8:** CSWinTT running on the target GPU server.

	AUC	*P*	$P_{\mathrm{normal}}$
**Org.**	90.04	88.63	91.47
**Sol.**	88.46	87.19	89.78

## Data Availability

Publicly available datasets were analyzed in this study. The data can be found in this link: https://tracking-net.org/ (accessed on 15 September 2022).
